# Days Not at Home: Association of Vulnerability with Healthcare Utilization After Hospitalization for Heart Failure

**DOI:** 10.1007/s11606-024-08872-x

**Published:** 2024-09-27

**Authors:** Sarah A. Welch, Chiara Di Gravio, Jonathan S. Schildcrout, Ricardo Trochez, Yaping Shi, Devika Nair, Eduard E. Vasilevskis, Amanda S. Mixon, Susan P. Bell, Sunil Kripalani

**Affiliations:** 1 Department of Physical Medicine & Rehabilitation, Vanderbilt University Medical Center, Nashville, TN USA; 2 Geriatric Research Education and Clinical Center, Tennessee Valley Healthcare System, Nashville, TN USA; 3 Department of Epidemiology and Biostatistics, School of Public Health, Imperial College London, London, UK; 4 Department of Biostatistics, Vanderbilt University Medical Center, Nashville, TN USA; 5 Division of General Internal Medicine and Public Health, Department of Medicine, Center for Clinical Quality and Implementation Research, Vanderbilt University Medical Center, Nashville, TN USA; 6 Division of Nephrology Medicine, Department of Medicine, Vanderbilt University Medical Center and Vanderbilt O’Brien Center for Kidney Disease, Nashville, TN USA; 7 Division of Hospital Medicine, School of Medicine and Public Health, University of Wisconsin, Madison, WI USA; 8 Vanderbilt Center for Health Services Research, Nashville, TN USA; 9 Center for Quality Aging, Vanderbilt University Medical Center, Nashville, TN USA; 10 Division of Geriatric Medicine, Department of Medicine, Vanderbilt University Medical Center, Nashville, TN USA; 11 Division of Cardiovascular Medicine, Department of Medicine, Vanderbilt University Medical Center, Nashville, TN USA; 12 Section of Hospital Medicine, Division of General Internal Medicine & Public Health, Vanderbilt University Medical Center, Nashville, TN USA; 13 Center for Musculoskeletal Research, Vanderbilt University Medical Center, Nashville, USA

**Keywords:** healthcare utilization, heart failure, hospitalization, vulnerability

## Abstract

**Background:**

Heart failure (HF) hospitalizations are characterized by vulnerability in functioning and frequent post-discharge healthcare utilization in both acute and post-acute settings.

**Objective:**

To determine, in patients hospitalized for decompensated HF, the association of vulnerability with (1) detailed forms of post-discharge healthcare utilization, and (2) days spent away from home after initial hospital discharge.

**Design:**

Secondary analysis of a prospective longitudinal cohort study from a single-center academic institution in the USA.

**Participants:**

Adults admitted with acute decompensated HF who were discharged alive.

**Main Measures:**

The Vulnerable Elders Survey 13 (VES-13) measured functional vulnerability at baseline. The primary outcome was the Highest Healthcare Utilization (HHU) 90 days post-discharge, from the following ordered categories: at home, emergency room visit, skilled nursing facility stay, hospital readmission, or death. The secondary outcome was the proportion of days not at home (DNAH) within the first 90 days. Analyses were performed using a partial proportional odds model with adjustment for demographics and health characteristics.

**Key Results:**

A total of 806 patients were included with median age 65, interquartile range [IQR] 55–73 years. Fewer than half (*N* = 345 [43%]) of patients remained alive and at home during 90-day follow-up. There were 286 [35%] hospital readmissions and 70 [8.7%] participants died. The median DNAH was 3 [IQR 0–16]. Increased vulnerability was associated with (1) HHU, (2) higher odds of utilizing healthcare or dying versus being at home alive 90 days post-discharge (OR 1.81 [95% CI, 1.35, 2.42]), and (3) higher odds of DNAH in the first 90 days (OR 1.55 [95% CI, 1.27, 1.89]).

**Conclusions:**

In this cohort of patients hospitalized for decompensated HF, vulnerability predicted higher levels of healthcare utilization, as well as total days not at home in the 90 days following hospitalization. Vulnerability may have clinical applications to identify patients at greatest need for comprehensive, patient-centered discharge planning.

**Supplementary Information:**

The online version contains supplementary material available at 10.1007/s11606-024-08872-x.

## INTRODUCTION

Heart failure (HF) exacerbations are the leading cause of hospitalizations in the United States, and their prevalence is expected to double in the next 40 years.^1,2^ The presence of vulnerability, a patient-reported measure of decreased functional status, is common in patients with HF surrounding the time of hospitalization.^3,4^ While vulnerability persists after the index hospitalization, the relationship between it and post-discharge healthcare consumption is not well described.

Meanwhile, the post-discharge period after HF hospitalization has gotten national attention as it accounts for a disproportionate amount of healthcare dollars spent.^5–8^ HF hospitalizations and readmissions are estimated to reach $2.8 billion by 2030.^9^ Studies focus on 30-day hospital readmissions and other binary quality and safety indicators important to hospitals despite known poor health-related quality of life following hospitalization for HF.^10^ Limiting measurement efforts to binary acute events post-discharge fails to tell the comprehensive story missing granular outcomes important to patients such as skilled nursing facility stays, emergency room visits, or multiple events.

Studies suggest that HF patients prioritize quality over quantity of life, and that chronically ill patients value connected time with family and friends, which usually takes place best at home.^11,12^ Recently, measures like healthy days at home have emerged as valuable patient-centered outcomes that reflect measurement of health during a cumulative period of defined time rather than just during acute events.^13^ However, there remains a paucity of studies reflecting this form of measurement in HF patients, the highest healthcare utilizers nationally.

The objective of the current study was to describe the epidemiology and patterns of post-discharge healthcare utilization in vulnerable patients hospitalized for heart failure. (1) We used a prospective longitudinal cohort of HF hospitalizations with 90 days of post-discharge follow-up to test whether vulnerability associated with ordinally ranked detailed forms of healthcare utilization called Highest Healthcare Utilization (HHU) including home, emergency room (ER) visit, skilled nursing facility (SNF) stay, rehospitalization, or death. (2) We also tested whether vulnerability associated with a cumulative period of days not at home (DNAH).

## METHODS

### Study Design

This is a secondary analysis of a prospective observational study designed to investigate the impact of social, behavioral, and functional determinants of health on post-discharge outcomes in adults hospitalized for cardiovascular disease. Participants were admitted between 2011 and 2015 at an academic tertiary care hospital. A detailed description of the study rationale, design, and methods is described elsewhere.^14^ It was approved by the University Institutional Review Board, and participants provided informed consent. The Strengthening the Reporting of Observational Studies in Epidemiology (STROBE) guidelines were followed for presentation of study findings. The original study collected discrete data on hospital and skilled nursing facility utilization after discharge.

### Study Cohort

**Figure 1:**
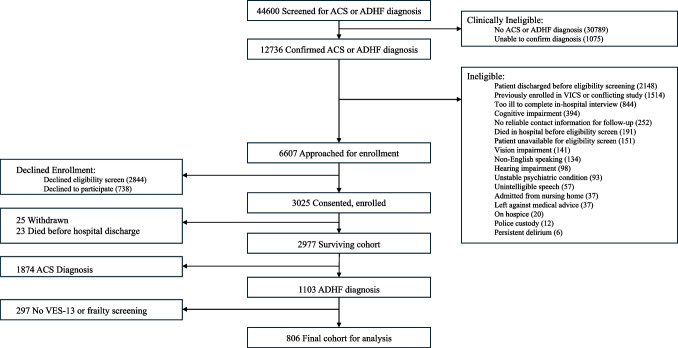
Study flow diagram. ACS, Acute Coronary Syndrome; ADHF, Acute Decompensated Heart Failure; VES-13, Vulnerable Elder Survey-13.

We included participants in this study who were hospitalized for acute decompensated heart failure (ADHF) as determined by a physician’s review of the medical record. Research assistants assessed for the presence of the following exclusion criteria: less than 18 years of age, non-English speaking, unstable psychiatric illness, a low likelihood of follow-up (e.g., no reliable telephone number), severe cognitive impairment (e.g., dementia documented in chart), on hospice, or otherwise too ill to complete an interview. Individuals that were too ill to consent or had delirium when initially approached were re-assessed for up to 7 days.^15,16^ A study flow diagram is included in Fig. 1. Participants completed a detailed in-person interview during enrollment in the hospital and were then followed up at approximately 3–5 days, 30 days, and 90 days after hospital discharge via telephone. Patients who died prior to discharge were excluded from the present analysis.

### Vulnerability

The primary predictor of interest was measured using the Vulnerable Elders Survey 13 (VES-13), a validated, 13-item, patient-reported measure of physical function.^17^ When assessing baseline vulnerability, patients were asked to recall functional status prior to hospital admission. Vulnerability is scored from 0 to 10 with 0–2 representing patients who are not vulnerable, 3–6 vulnerable, and 7–10 extremely vulnerable.^3^ The VES-13 has predictive validity (*C*-statistic 0.78) for functional decline and mortality.^18,19^

### Covariates

Participants completed an in-person interview-administered baseline assessment composed of demographic information including gender, self-reported race, educational attainment, marital status, employment status, and household income. Hospitalization and clinical characteristics abstracted from the medical record included hospital length of stay, number of hospitalizations in the past 12 months, body mass index (BMI), medical history of HF, HF subtype (classified by left ventricular ejection fraction [LVEF]), coronary artery disease, chronic obstructive pulmonary disease (COPD), and diabetes mellitus (DM). Comorbidity burden was summarized using van Walraven’s scoring of the Elixhauser comorbidity list, which is calculated using ICD codes and is based on the presence or absence of 30 comorbidities.^20,21^

### Highest Healthcare Utilization

Daily data were collected via patient self-report and validated through comprehensive review of medical records, including records from outside facilities. There was no loss to follow-up or missing data. The primary outcome Highest Healthcare Utilization (HHU) was a composite, ordinal measure which synthesized daily healthcare utilization data across the first 90 days after discharge. Daily post-discharge healthcare utilization for each patient was coded into one of five HHU states, ordered from the best to the worst outcome: at home, emergency room (ER) visit without hospitalization, skilled nursing facility (SNF) stay, rehospitalized, or not alive. Because an ER visit typically consists of a transient interaction, it was ordered as the second best outcome. Since acute care hospitals usually provide more intensive services and treat more complex medical needs, rehospitalization was ordered higher than SNF. For each patient, the single worst state across the first 90 days after discharge was assigned as the primary outcome. For example, an outcome was coded as an “ER visit” if the visit did not result in hospitalization; an outcome was coded as a “SNF stay” if the patient spent at least once night at a SNF, but neither rehospitalization nor death occurred; and outcome was coded as a hospitalization if a patient spent at least one night in the hospital without dying.

### Days Not at Home

The secondary outcome was days not at home (DNAH), which was measured as a proportion of days (out of 90) not spent at home alive during the follow-up period. To calculate DNAH, we added the number of days spent in each healthcare utilization setting out of 90. For example, a patient who spent 4 days readmitted to the hospital and 5 days in a SNF would have 9 days of utilization; DNAH would be calculated as 9/90, or 0.10. We also incorporated multiple events and mortality during the 90-day period. For example, a patient who had a 5-day rehospitalization, another 6-day rehospitalization, and died on day 72 would have 29 days not at home alive; and DNAH would be calculated as 29/90, or 0.32.

### Statistical Analysis

Demographic and baseline characteristics for the study sample are described with the median and interquartile range (IQR) for continuous variables and with a frequency and percent for discrete variables. To study the relationship between VES-13 and the ordered primary outcome variable, HHU, we used cumulative logistic regression analyses while controlling for covariates described earlier. We chose proportional odds (PO) logistic regression because it models the (log) odds for worse outcomes associated with changes in exposure values. To study the association of vulnerability with HHU, we relaxed the PO assumption and fit a partial proportional odds (PPO) model with PO imposed on covariate associations with HHU. This allowed for greater flexibility in the functional form of the association of VES-13 with HHU. By fitting the PPO model, we also estimated distinct associations for the relationship between VES-13 and the following dichotomizations of HHU outcomes that increased in severity: (1) ER visit, SNF, hospitalized or death, versus at home; (2) SNF, hospitalized, or death versus ER visit or at home; (3) hospitalized or death versus SNF, ER visit or at home; and (4) death versus hospitalized, SNF, ER visit, or home. Odds ratios and 95% confidence intervals characterized the relationship between VES-13 and each of the binary outcome variables, all of which are included in the PPO model.

To study the relationship between the proportion of DNAH and VES-13 during the 90-day follow-up period, we fit a covariate-adjusted, beta-binomial logistic regression model. Odds ratios and 95% confidence intervals quantified the strength and uncertainty of observed associations. The beta-binomial logistic regression model retains the interpretation of standard logistic regression, but appropriately acknowledged overdispersion in the DNAH response. It modeled the (log) odds of being “not at home” across all days, or for a representative day across the 90-day follow-up period. We modeled VES-13 as a linear and continuous variable, and for all models we characterize effect size for VES-13 and other continuous variables using an interquartile range change.

Because VES-13 incorporates age into its score, age was left out of the primary models. Models were constructed at the time participants were discharged, and included length of stay, a potential mediator. Sensitivity analyses that adjusted for age and removed length of stay were constructed and are shown in the Supplementary Information. Across all participants and variables, 1.11% of data were missing, and 21.56% of participants had at least some missing data. Missing data were imputed with a predictive mean matching algorithm. Multiple imputation analyses were conducted, and results were summarized across ten imputed datasets. All analyses were conducted using R v.4.3.0, and specifically the mice and VGAM packages.^22–24^

## RESULTS

A total of 806 patients were included with median [interquartile (IQR)] age, 65 [55–73] years; 445 [55%] were male; and most [644 (80%)] were self-reported white (Table 1). The median [IQR] initial hospital length of stay was 5 [3–8] days with moderate risk Elixhauser scores, median [IQR] 15 [9–21]. The patients were mostly vulnerable [525 (65%)] with median [IQR] VES-13 scores 3 [2–7] where 3 or more represents vulnerability. There were 345 (43%) patients who remained alive and at home throughout the 90 days of follow-up post-discharge. The other Highest Healthcare Utilizations included ER visit [*n* = 69, (8.6%)], SNF [*n* = 36 (4.5%)], hospital readmission [*n* = 286 (35%)], and death [*n* = 70 (8.7%)]. The median [IQR] number of days not at home was 3 (0–16).

**Table 1:** Patient Characteristics

Characteristic	Overall* n = 806	Not vulnerable* (VES-13 = 0–2) *n* = 279	Vulnerable* (VES-13 = 3–10) *n* = 525
Age, years	65 (45,55,73,80) (64,14)	64 (41,53,71,76) (61,14)	66 (47,56,75,83) (65,14)
Gender			
Female	361 (45%)	102 (37%)	259 (49%)
Male	445 (55%)	177 (63%)	266 (51%)
Race			
African American	138 (17%)	49 (18%)	89 (17%)
Other	24 (3.0%)	8 (2.9%)	16 (3.0%)
White	644 (80%)	222 (80%)	420 (80%)
Years of education	13 (11,12,16,18) (13.6, 3.1)	14 (11,12,16,18) (13.99, 3.10)	12 (10,12,15,18) (13.31, 3.02)
Employment status			
Employed	185 (23%)	109 (39%)	76 (14%)
Unemployed	27 (3.3%)	10 (3.6%)	17 (3.2%)
Retired	372 (46%)	120 (43%)	250 (48%)
Unable to work (disabled)	222 (28%)	40 (14%)	182 (35%)
Annual household income			
Less than $10,000	64 (8.5%)	23 (8.7%)	41 (8.5%)
$10,000 to < $15,000	67 (8.9%)	16 (6.1%)	51 (11%)
$15,000 to < $20,000	71 (9.5%)	16 (6.1%)	55 (11%)
$20,000 to < $25,000	81 (11%)	22 (8.3%)	58 (12%)
$25,000 to < $35,000	121 (16%)	38 (14%)	83 (17%)
$35,000 to < $50,000	133 (18%)	51 (19%)	82 (18%)
$50,000 to < $75,000	93 (12%)	38 (14%)	54 (11%)
$75,000 to < $100,000	57 (7.6%)	29 (11%)	28 (5.8%)
$100,000 or more	63 (8.4%)	31 (12%)	32 (6.6%)
(Missing)	56	15	41
Marital status			
Married/living with partner	374 (46%)	120 (43%)	254 (48%)
Separated/widowed/single/never married	432 (54%)	159 (57%)	271 (52%)
Elixhauser score	15 (4,9,21,28) (16,9)	13 (4,8,21,27) (15,9)	16 (5,10,22,29) (16,9)
Body mass index (kg/m^2^)	29 (22,25,35,41) (31,8)	29 (22,25,34,40) (30,7)	30 (22,26,36,42) (32,9)
Hospitalizations in past 12 months, number	1.00 (0,0,3,5) (1.96, 2.28)	1.00 (0,0,2,3) (1.19, 1.56)	2.00 (0,1,4,5) (2.37, 2.48)
Length of stay (days)	5 (2,3,8,14) (6.8, 6.4)	4 (2,3,7.5,11.2) (6.3, 6.6)	5 (2,3,8,15) (7.1, 6.3)
Ejection fraction			
Borderline (between 40 and 50)	59 (7.4%)	24 (8.9%)	34 (6.6%)
Low (less than or equal to 40)	394 (50%)	144 (53%)	249 (48%)
Preserved (greater than or equal to 50)	339 (43%)	103 (38%)	236 (45%)
(Missing)	14	8	6
Vulnerability score (VES-13)	3 (1,2,7,7)* (4.05, 2.74)	1 (0,0,2,2) (1.06, 0.78)	7 (3,3,7,7) (5.64, 1.97)

^*^Median (10%, 25%, 75%, 90%) (mean, SD); *n* (%), *VES-13 missing for *n* = 2

**Figure 2:**
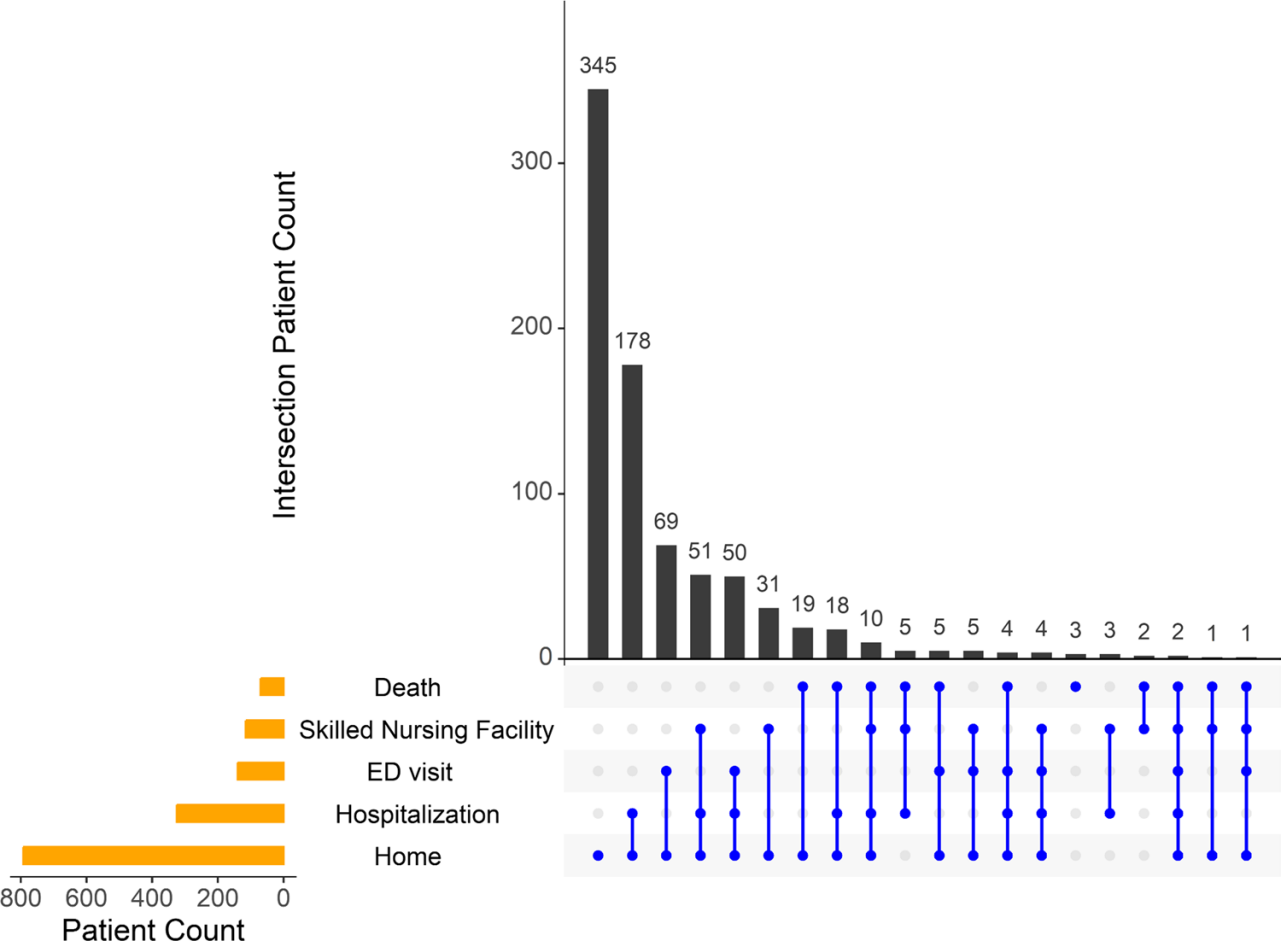
Prevalence of post-discharge healthcare utilization. Prevalent combinations of post-discharge healthcare utilization trajectories include home, hospital readmission, ED visits, skilled nursing facilities, and/or death. Each possible combination is defined on the *x* axis (dot defines all discharge outcomes for each group) against prevalence of that outcome on the *y* axis. The horizontal bars show the marginal frequencies of healthcare utilization including “Home.” *ED, Emergency Department.

There were a total of 20 possible combinations of healthcare utilization shown in Fig. 2. Following the most common utilization, home, the second most common utilization combination was to discharge home with at least one rehospitalization, occurring in 178 patients (22%). There were 2 patients who went home and also had one each of every other possible healthcare utilization before death (at least one ER visit, rehospitalization, and SNF stay).

**Figure 3:**
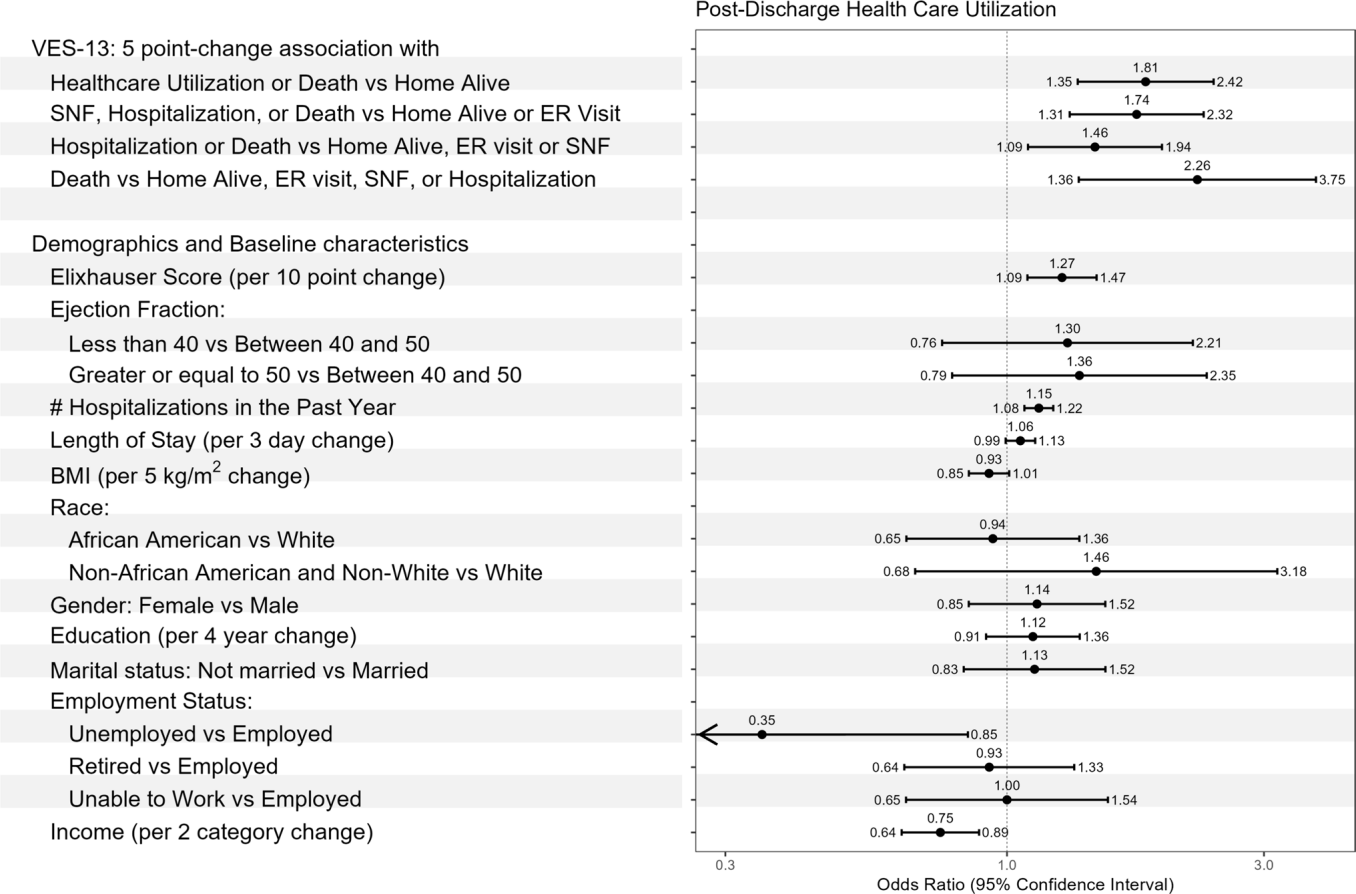
Vulnerability and Highest Healthcare Utilization. Estimates of association (odds ratio, 95% CI) between the Vulnerable Elder Survey-13 (VES-13) score and Highest Healthcare Utilization (HHU) using the partial proportional odds model. A 5-point change in VES-13 was specified to quantify its effect on HHU as a score of 5 points on the VES-13 represents the effect per one IQR change in VES-13. Other covariates included Elixhauser score, ejection fraction, number of hospitalizations in the past year, length of hospital stay, Body Mass Index (BMI), race, gender, education, marital status, employment status, and income. *SNF, Skilled Nursing Facility; ER, Emergency Room.

When healthcare utilization was simplified into five HHU states and PPO logistic regression models were constructed, vulnerability was consistently associated with higher forms of healthcare utilization and death during the 90 days post-discharge (Fig. 3). A 5-point increase (an IQR change) in VES-13 was associated with 81% higher odds of healthcare utilization or death in the first 90 days versus being at home alive [OR 1.81 (95% CI, 1.35, 2.42)]. Further, a 5-point increase in VES-13 was associated with 126% higher odds of dying in the first 90 days [OR 2.26 (95% CI, 1.36–3.75)].

**Figure 4:**
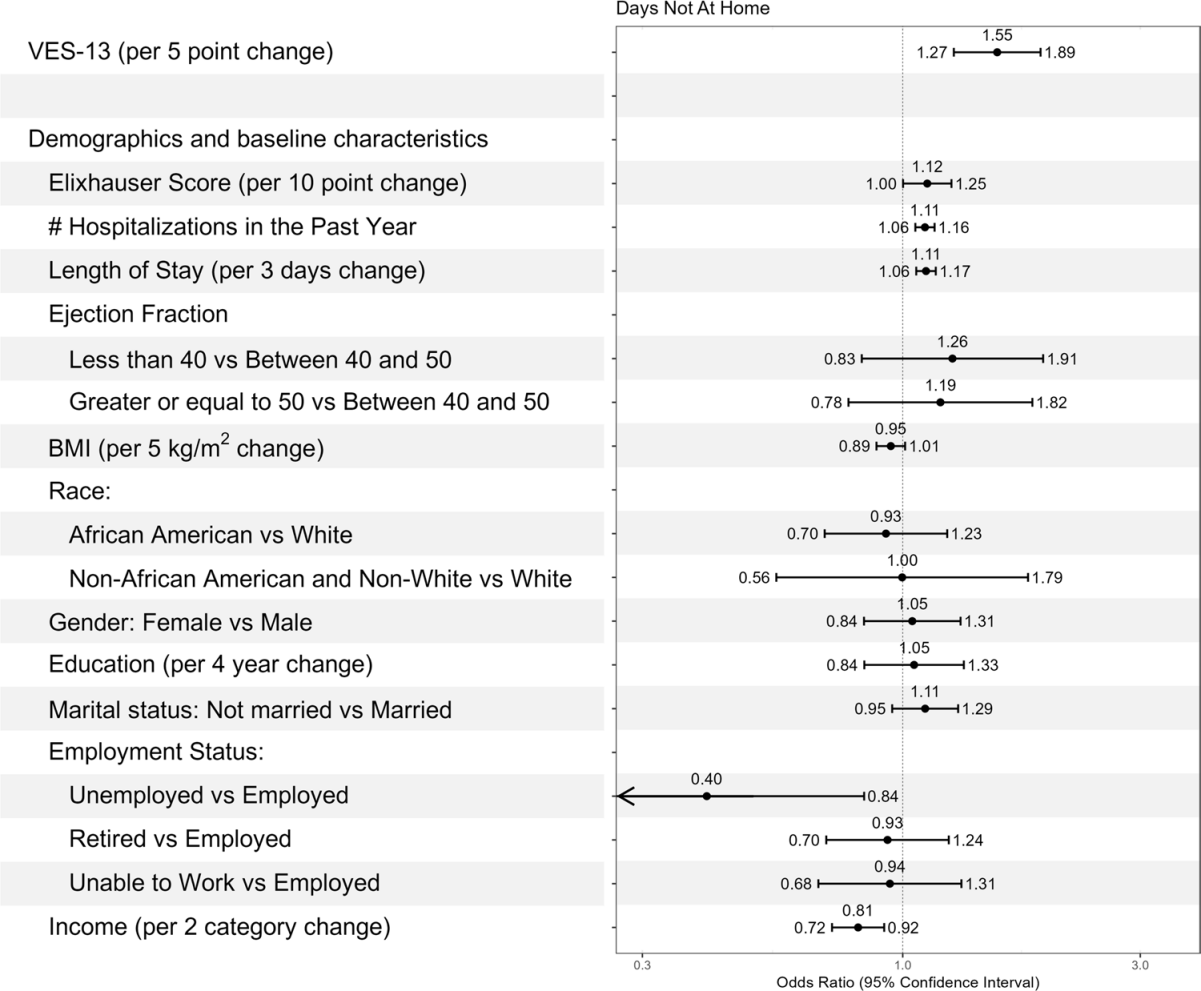
Vulnerability and Days Not at Home. Estimates of association (odds ratio, 95% CI) between the Vulnerable Elder Survey-13 (VES-13) score and the proportion of Days Not At Home (DNAH) using the beta-binomial logistic model. A 5-point change in VES-13 was specified to quantify its effect on DNAH as a score of 5 points on the VES-13 represents the effect per one IQR change in VES-13. Other covariates included Elixhauser score, number of hospitalizations in the past year, length of hospital stay, ejection fraction, Body Mass Index (BMI), race, gender, education, marital status, employment status, and income.

Vulnerability was also independently associated with days not at home. There was a 55% increase in the odds of not being at home each day per 5-point increase in VES-13 [OR 1.55 (95% CI, 1.27–1.89)] (Fig. 4). Covariate associations with HHU and DNAH are also shown in Figs. 3 and 4, but should be interpreted with caution since considerations of the potential for confounding focused on VES-13 but not the covariates. Sensitivity analyses that adjusted separately for age and removed length of stay did not change the direction or magnitude of the association between VES-13 and post-discharge healthcare utilization or days not at home (Supplementary Information).

## DISCUSSION

In the 90 days after discharge from hospitalization for HF, we found that 70 (8.7%) patients died, while 286 (35%) experienced a readmission, 36 (4.5%) a SNF stay, and 69 (8.6%) an ER visit. Furthermore, we found that vulnerability predicted higher forms of healthcare utilization and days not at home. This study contributes to a growing body of evidence for patients hospitalized for HF, a high-risk group, during a crucial post-discharge time when mortality, readmissions, and impaired quality of life are common.^25,26^

Although hospital readmissions are an important quality indicator, our detailed characterization which demonstrated 20 different combinations of healthcare utilization (Fig. 2) may better contextualize patient experience, and reflect patient-centered outcomes.^5,13,27^ In our study, the most prevalent healthcare utilization trajectory was characterized by patients who went home but also had at least one hospital readmission depicted in Fig. 2. The 90-day readmission rate was 35%, which is similar to the reported national average of 32%.^27^ The second most common trajectory included 69 (8.6%) patients who went home but also had an ER visit. In other studies, ER visits are often excluded or combined with readmissions compositely, though they are not equivalent, and are notably undesirable outcomes for patients.^28^ The third most common trajectory included 51 (6.3%) patients with at least two healthcare utilization interactions including (1) a SNF stay and (2) a hospital readmission. Of note, certain exclusion criteria for the main study (e.g., admission from a nursing home, moderate to severe cognitive impairment, or not providing informed consent) likely contributed to a lower percentage of patients being discharged to SNF. Though our data does not capture the ordering of these events, future work should look at risk stratification of such trajectories. Detailed consideration of outcomes such as this is important as HF post-acute care utilization is on the rise and can represent an important marker for patient functioning and health.^29^

Our study is also novel in creating a HHU combined ordinal outcome which included home, ER visit, SNF stay, rehospitalization, and death. The HHU ordering of best to worst was created based on acuity of care (e.g., skilled nursing facility stay less acute than a rehospitalization) and temporality (e.g., ER visit less duration than a skilled nursing facility stay). The measurement of DNAH in Fig. 4 added sensitivity, providing a cumulative measure of outcomes in the 90 days post-discharge period.^30^ DNAH accounts for repeated hospitalizations, length of stay, and time spent in SNFs. Furthermore, it has been identified as an important patient-centered outcome. Decreased home time has been associated with worse outcomes such as self-rated health, low social activity, and depression.^31^ This is not surprising as studies suggest that patients with HF prioritize quality over quantity of life.^11^

To our knowledge, this is the first study to demonstrate that vulnerability is a potent predictor of many types of post-discharge healthcare utilization and increasing days spent away from home. Improved identification of vulnerable hospital patients and targeted interventions are needed to increase the time that patients spend at home and improve their quality of life. The VES-13 was initially studied in the outpatient setting and used as a quick measure to identify vulnerable patients at risk of functional decline or death.^3^ More recent work showed that inpatient VES-13 implementation was feasible, and hospital patients with higher vulnerability scores also experienced prior hospitalizations.^4^ Because the VES-13 is a patient self-report tool that includes some functional limitation measures, components of it have potential for inpatient intervention. Our data suggest that an inpatient VES-13 assessment may have utility for stratifying patients at risk for HHU, as well as DNAH. Although there is not a gold standard for hospital functional status or frailty, the VES-13 should be compared against other measures, and future work should examine appropriate actions to modify risk during index hospitalization and discharge planning. For example, communication among teams to prioritize high-risk patients (scores of 3+) for early follow-up, telehealth appointments, and access to resources such as home health, physical therapy, occupational therapy, or cardiac rehabilitation could be investigated. Future investigations might also consider exploring whether the length of time from discharge to scheduled follow-up contributes to the model as a potential target for intervention.

Limitations of the study included performance at a single site large academic medical center with tertiary referral care for HF which may limit generalizability. VES-13 measurement was collected via patient report, creating the possibility of bias as participants may under- or overestimate their own health. Additionally, patients with cognitive impairment or other chronic conditions limiting their ability to consent were excluded from the study, potentially excluding some of the most vulnerable patients, yet vulnerability was still a common phenotype in the study. This study also had many strengths. It is a large, well-characterized, prospective cohort that included a 90-day post-discharge follow-up with detailed data collected on healthcare utilization. It used the VES-13, a validated measure of function with predictive validity for functional decline and mortality.

## CONCLUSIONS

In this cohort of patients hospitalized for decompensated HF, there were 20 total possible combinations of healthcare utilization in the 90 days after discharge. Vulnerability was predictive of higher forms of post-discharge healthcare utilization and days not at home. Vulnerability may have clinical application to identify patients at greatest need for comprehensive patient-centered discharge planning and follow-up.

## Supplementary Information

Below is the link to the electronic supplementary material.

Supplementary File 1. Vulnerability and Highest Healthcare Utilization With Age and Without Length of Stay Included in the Model. Estimates of association (odds ratio, 95% CI) between the Vulnerable Elder Survey-13 (VES-13) score and Highest Healthcare Utilization (HHU) using the partial proportional odds model. A 5-point change in VES-13 was specified to quantify its effect on HHU as a score of 5 points on the VES-13 represents the effect per one IQR change in VES-13. Other covariates included age, Elixhauser score, ejection fraction, number of hospitalizations in the past year, length, Body Mass Index (BMI), race, gender, education, marital status, employment status, and income. *SNF = Skilled Nursing Facility, ER= Emergency Room. (PNG 699 KB)

Supplementary File 2 Vulnerability and Days Not at Home, With Age and Without Length of Stay Included in the Model. Estimates of association (odds ratio, 95% CI) between the Vulnerable Elder Survey-13 (VES-13) score and the proportion of Days Not at Home (DNAH) using the beta-binomial logistic model. A 5-point change in VES-13 was specified to quantify its effect on DNAH as a score of 5 points on the VES-13 represents the effect per one IQR change in VES-13. Other covariates included age, Elixhauser score, number of hospitalizations in the past year, ejection fraction, Body Mass Index (BMI), race, gender, education, marital status, employment status, and income. (PNG 610 KB)

## Data Availability

The data that support the findings of this study are available from the corresponding author, SW, upon reasonable request.
