# Supplementary figures and images for: Days Not at Home: Association of Vulnerability with Healthcare Utilization After Hospitalization for Heart Failure
Source: J Gen Intern Med. 2024 Sep 27;40(3):547–55. doi: 10.1007/s11606-024-08872-x (PMC11861822; doi:10.1007/s11606-024-08872-x)

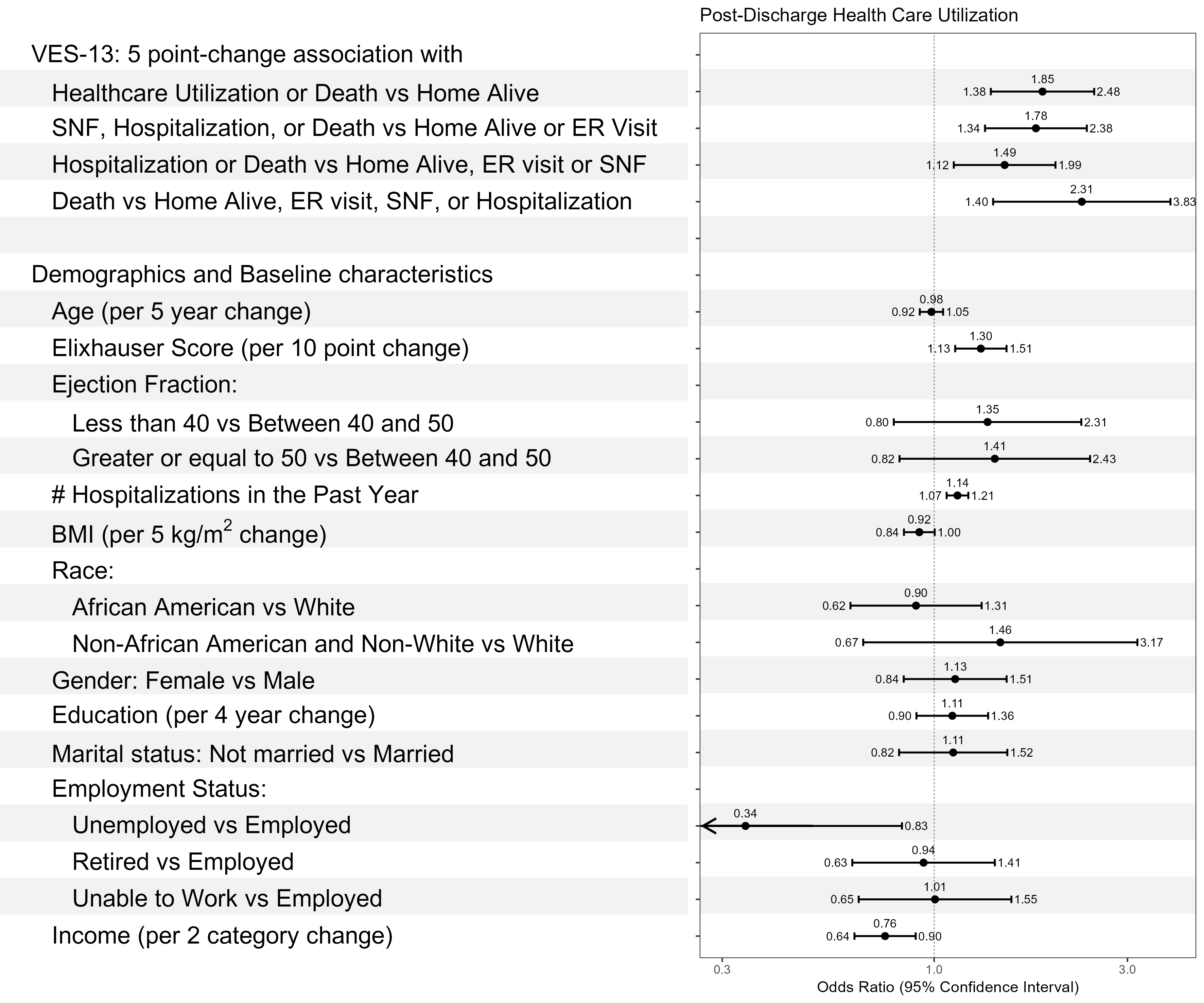

Supplement: Supplementary file 1 — Vulnerability and Highest Healthcare Utilization With Age and Without Length of Stay Included in the Model. Estimates of association (odds ratio, 95% CI) between the Vulnerable Elder Survey-13 (VES-13) score and Highest Healthcare Utilization (HHU) using the partial proportional odds model. A 5-point change in VES-13 was specified to quantify its effect on HHU as a score of 5 points on the VES-13 represents the effect per one IQR change in VES-13. Other covariates included age, Elixhauser score, ejection fraction, number of hospitalizations in the past year, length, Body Mass Index (BMI), race, gender, education, marital status, employment status, and income. *SNF = Skilled Nursing Facility, ER= Emergency Room. (PNG 699 KB) [file 11606_2024_8872_MOESM1_ESM.png]

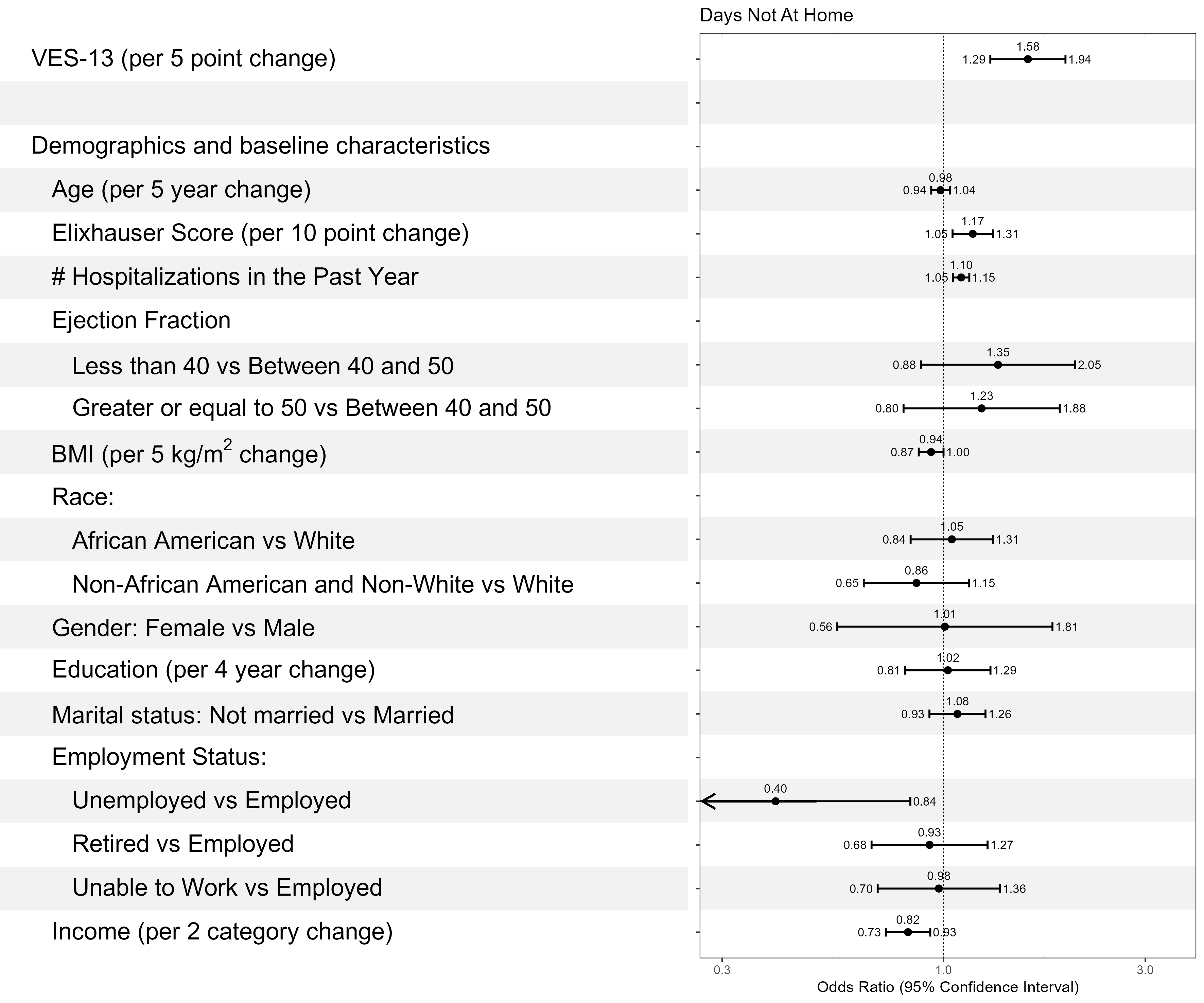

Supplement: Supplementary file 2 — Vulnerability and Days Not at Home, With Age and Without Length of Stay Included in the Model. Estimates of association (odds ratio, 95% CI) between the Vulnerable Elder Survey-13 (VES-13) score and the proportion of Days Not at Home (DNAH) using the beta-binomial logistic model. A 5-point change in VES-13 was specified to quantify its effect on DNAH as a score of 5 points on the VES-13 represents the effect per one IQR change in VES-13. Other covariates included age, Elixhauser score, number of hospitalizations in the past year, ejection fraction, Body Mass Index (BMI), race, gender, education, marital status, employment status, and income. (PNG 610 KB) [file 11606_2024_8872_MOESM2_ESM.png]
